# Growth impairment and limited range of joint motion in children should raise suspicion of an attenuated form of mucopolysaccharidosis: expert opinion

**DOI:** 10.1007/s00431-019-03330-x

**Published:** 2019-02-11

**Authors:** Nathalie Guffon, Pierre Journeau, Anaïs Brassier, Juliane Leger, Bertrand Chevallier

**Affiliations:** 1 Reference center of Inherited Metabolic disorder, CERLYMM, Département de Pédiatrie, HCL Hopital Femme Mère Enfant, 59 Boulevard Pinel, 69677 Bron cedex, France; 2 Paediatric Orthopaedic Surgery Department, Lorraine University Hospital Centre, Children’s Hospital, Vandoeuvre lès Nancy, France; 3 Reference Center of Inherited Metabolic Diseases, Imagine Institute, Hospital Necker Enfants Malades, APHP, University Paris Descartes, Paris, France; 4 Assistance Publique-Hôpitaux de Paris, Robert Debré University Hospital, Pediatric Endocrinology Diabetology Department, Reference Centre for Endocrine Growth and Development Diseases, Paris Diderot University, Sorbonne Paris Cité, F-75019 Paris, France; 5 Groupe de Pédiatrie Générale - Société Française de Pédiatrie, Boulogne-Billancourt, Department of Pediatrics and Pediatric Emergency, Ambroise-Paré Hospital, Boulogne-Billancourt, France

**Keywords:** Attenuated mucopolysaccharidosis, Glycosaminoglycans, Lysosomal enzyme deficiency, Growth impairment, Delayed puberty, Dysostosis multiplex, Joint contractures, Early diagnosis, Enzyme replacement therapy, Algorithm

## Abstract

Growth impairment together with bone and joint involvement is common to most patients with mucopolysaccharidosis (MPS) disorders. The genetic basis for these metabolic disorders involves various enzyme deficiencies responsible for the catabolism of glycosaminoglycans (GAGs). The incomplete degradation and subsequent accumulation of GAGs result in progressive tissue damage throughout the body. Bone ossification is particularly affected, with the consequent onset of dysostosis multiplex which is the underlying cause of short stature. Joint manifestations, whether joint contractures (MPS I, II, VI, VII) or hyperlaxity (MPS IV), affect fine motor skills and quality of life. Subtle decreases in growth velocity can begin as early as 2–4 years of age. Pediatricians are in the front line to recognize or suspect MPS. However, given the rarity of the disorders and variable ages of symptom onset depending on disease severity, recognition and diagnostic delays remain a challenge, especially for the attenuated forms. Prompt diagnosis and treatment can prevent irreversible disease outcomes.

*Conclusion*: We present a diagnostic algorithm based on growth velocity decline and bone and joint involvement designed to help pediatricians recognize early manifestations of attenuated forms of MPS. We illustrate the paper with examples of abnormal growth curves and subtle radiographic nuances.

**Table:** 

**What is Known:** *• As mucopolysaccharidoses (MPSs) are rare genetic disorders infrequently seen in clinical practice, there can be a lag between symptom onset and diagnosis, especially of attenuated forms of the disease.* *• This highlights the need for increased disease awareness to recognize early clinical signs and subsequently initiate early treatment to improve outcomes (normal height potential) and possibly prevent or delay the development of irreversible disease manifestations.*	
**What is New:** *• Growth impairment co-presenting with limited range of joint motion and radiographic anomalies in children should raise suspicions of possible attenuated MPS (AMPS).* *• Experts present a diagnostic algorithm with detailed focus on the decline in growth velocity, delayed puberty and limitation in joint mobility seen in children with AMPS, to shorten time-to-diagnosis and treatment and potentially improve patient outcome.*

## Introduction

Mucopolysaccharidoses (MPSs) are rare metabolic disorders affecting between 3.4 and 4.5 per 100,000 live births [2]. They are caused by specific enzyme deficiencies responsible for the catabolism of glycosaminoglycans (GAGs), long-chain polysaccharides linked to a protein core which constitute the ground substance of connective tissues. The incomplete degradation of GAGs leads to their accumulation in cells and tissues resulting in increasing damage and clinical manifestations throughout the body [23, 34]. Seven clinical subtypes of MPS have been described: MPS I (Hurler, Hurler-Scheie, Scheie), MPS II (Hunter), MPS III (Sanfilippo), MPS IV (Morquio), MPS VI (Maroteaux-Lamy), MPS VII (Sly syndrome), and MPS IX caused by 11 enzyme deficiencies. As with most genetic disorders, there is a continuous spectrum of phenotypic expression from the very severe to the mild or attenuated with variable ages of onset and rates of progression. Nonetheless, most of the MPSs (I, II, IV, VI, and VII), while clinically distinct, present with bone and joint involvement, delayed puberty, and growth velocity decline [23, 25]. Other clinical manifestations include respiratory, cardiac, hearing, and visual impairments [29, 41, 42, 52].

Given the wide spectrum of clinical manifestations, recognition and correct diagnosis of MPSs are a challenge, especially for the attenuated forms (AMPSs) where phenotypes are subtle and slow progressing and appear later in childhood compared with the more severe forms of MPSs.

Once a clinical suspicion of MPS has been raised, both qualitative and quantitative assays of urinary GAG (uGAG) levels as well as concomitant enzyme activity assay in leukocytes or fibroblasts should be done to confirm the diagnosis [7, 23]. It should be noted that although uGAG concentrations are thought to correlate with disease severity, as uGAG levels decrease naturally with age, normal uGAG concentrations do not necessarily rule out a diagnosis of an AMPS especially in the older child or adolescent [7, 23]. Comprehensive uGAG testing and enzyme assays are complex and should be validated by a geneticist or metabolic specialist.

Treatment options are for the most part symptom-based palliative care. Hematopoietic stem cell transplantation (HSCT) is the mainstay treatment option for the more severe form of MPS I, showing effectiveness in prolonging survival, and slowing disease progression and cognitive decline in very young children [11, 13, 24, 39]. However, not all MPS types have shown benefit from HSCT and clinical experience is limited: it is currently being studied for severe MPS II in controlled clinical trials involving very young patients, and a few patients with MPS IV, mainly in Japan, have undergone this therapeutic option [4, 50, 57]. Enzyme replacement therapy (ERT) is recommended in the attenuated phenotypes and is currently available for MPSs I, II, IV, VI, and VII; patients benefit most if treatment is initiated in the early stages of the disease before irreversible tissue damage takes place [8, 14, 18, 21, 32]. Furthermore, ERT has been shown to positively influence growth (AMPSs I, II) if treatment is initiated before puberty [18, 20, 21, 47].

Despite recent advances in treatment therapies and management options, MPS patients, especially those with attenuated forms, can remain undiagnosed for years—even decades [9, 10, 19, 51]. The median age of onset of clinical features varies widely (as do presenting signs and symptoms) from one MPS type to another, ranging from 1.5 to 5.3 years for AMPS I to 2 to 7 years for AMPS II and 8 years for AMPS VI. The median age at diagnosis also varies widely, ranging from 4.9 to 9.6 years for AMPS I to 4 to 14 years for AMPS II [3, 5, 10, 19, 45, 53, 56].

Lack of familiarity with the disease awareness and poor clinical recognition are the main reasons for delayed diagnosis [5]. AMPS patients with unresolved symptoms are often referred to multiple specialists (hospital pediatricians, pneumologists, rheumatologists, orthopedic surgeons, endocrinologists, and geneticists) before a successful diagnosis is made [5]. Even among specialists, prompt and early diagnosis remains a challenge; MPSs are so rare that the likelihood of a pediatrician seeing a patient with AMPS in consultation is very low. The broad spectrum of phenotypic presentation, which may or may not have obvious physical or radiologic supporting evidence, further adds to the diagnostic challenge [7, 10, 16, 19]. A recent survey conducted to gauge clinical familiarity (recognition and diagnosis) with AMPS I among pediatricians and rheumatologists revealed that fewer than 40% of specialists were familiar with the disorder [5].

**Fig. 1:**
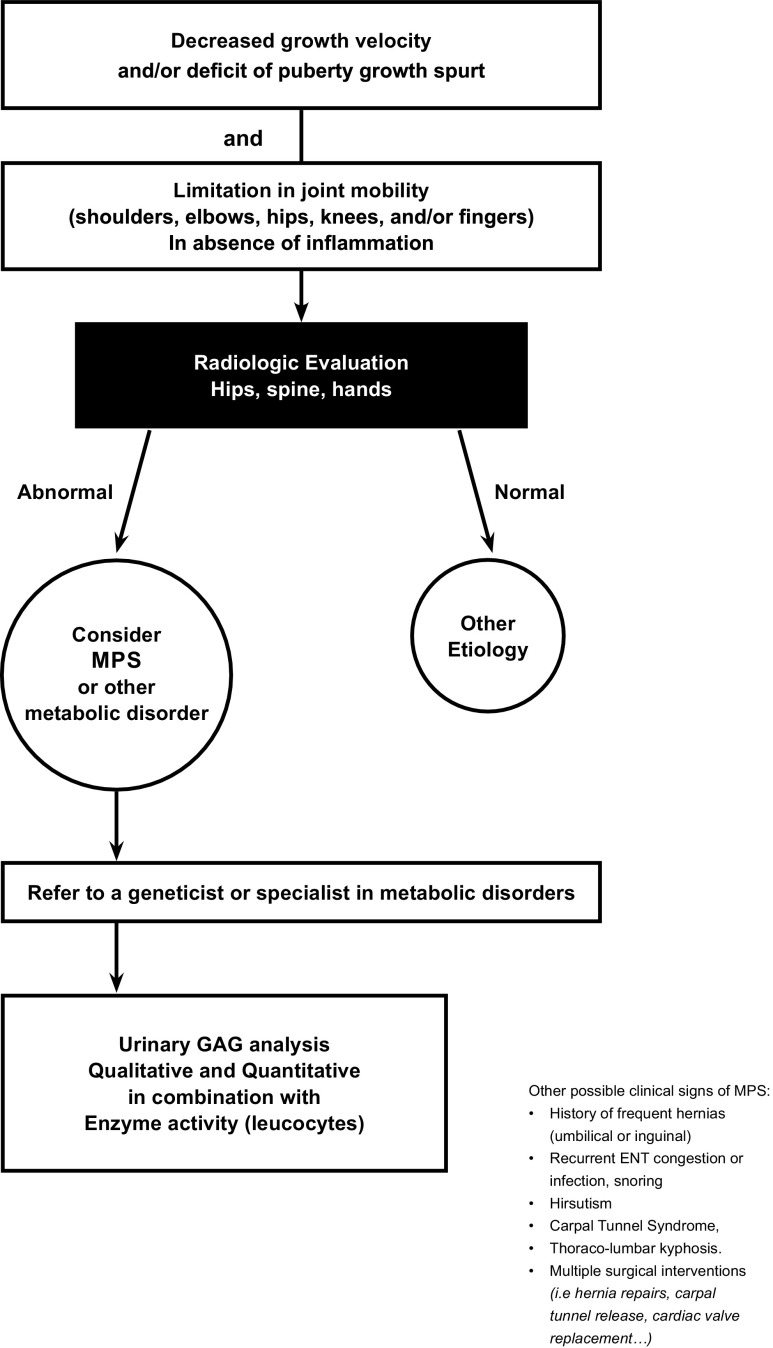
Diagnostic algorithm for attenuated mucopolysaccharidoses (AMPS). This diagnostic algorithm starts with growth problems or short stature. This algorithm can be used for attenuated forms of MPSs I, II, IV, VI, VII, and X. Limitations in joint mobility including limited extension; diminished abduction and anteflexion in shoulders; flexion deformity in elbows in AMPSs I, II, VI, and VII; ligamentous laxity bone hypoplasia; and joint hypermobility in the AMPS IV. Other signs, preceding symptoms which may or may not be apparent during physical examinations, including multiple and often unrelated surgical procedures occurring at atypical ages, compared with the general pediatric population. ENT: ear, nose, and throat. Other metabolic disorders: mucolipidosis. A correct diagnosis and confirmation of AMPS are complex and we recommend that once there is a suspicion of AMPS, the patient be referred to a geneticist or specialist in metabolic disorders for comprehensive GAG profile. We recommend both quantitative and qualitative (GAG profile) analyses in combination with leucocyte enzyme activity. False negatives can occur with spot screening

In view of the diagnostic challenges of AMPS, a more detailed insight into the clinical presentation and natural history of patients with AMPS might promote recognition, and accurate and early diagnosis of the disorders among pediatricians. Therefore, a group of five French pediatric specialists (in orthopedics, rheumatology, endocrinology, and inherited metabolic disorders) with expertise in the management of MPS or rare pediatric diseases convened to review and discuss AMPS clinical cases, clinical practices, and current knowledge of AMPS. These discussions resulted in preliminary recommendations to help pediatricians recognize the subtle manifestations of AMPSs, including idiopathic growth impairment in the context of other bone and joint manifestations and a constellation of non-specific signs and symptoms. A diagnostic algorithm is presented (Fig. 1). It should be noted that the algorithm does not cover AMPS III as its clinical presentation is distinctly different.

## Expert review and opinion

### Overview of growth profiles in AMPS patients

**Fig. 2:**
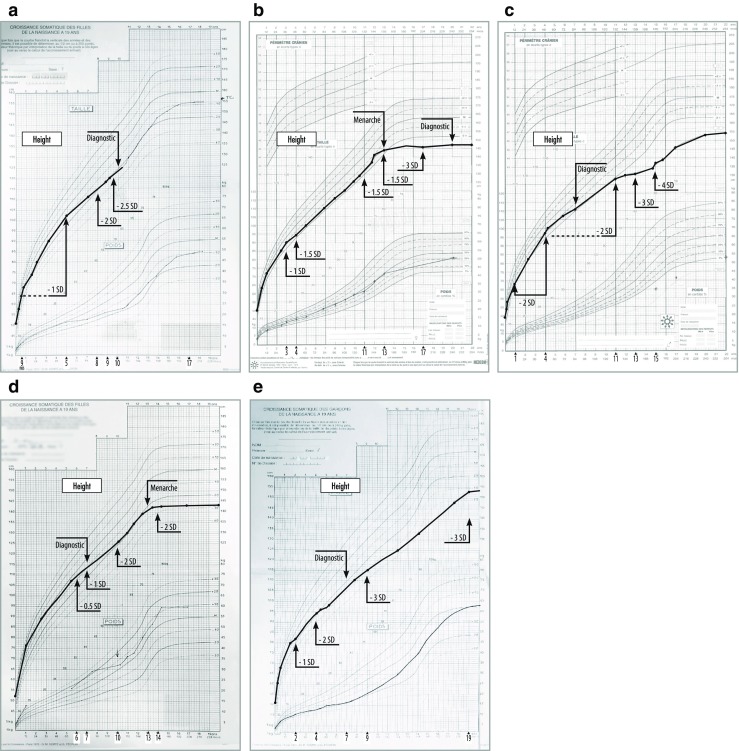
Growth charts from birth for individual patients with attenuated forms of MPS (I, II, IV, VI). SD, standard deviation; ERT, enzyme replacement therapy; DX, diagnosis. **a** Female patient with attenuated form of MPS I (Hurler-Scheie) shows normal growth until age of 9 months. Growth rate begins to progressively decline (− 1 SD until the age of 5 years; − 2 SD at 9 years; − 2.5 SD at 10 years). A diagnosis of Hurler-Scheie was confirmed at 10 years of age (123 cm, − 2 SD). **b** Female patient with attenuated form of MPS I (Scheie) shows normal growth until the age of 1, after which growth rate begins to progressively decline (− 1 SD at 3 years, − 1.5 SD at 4 years). Growth ceased at 13 years of age with an adult height of 146 cm (− 3 SD) at 17 years (target height 155.5 cm). A diagnosis of Scheie was not confirmed until 20 years of age. A slight pubertal growth gain of 10 cm was measured between 11 and 12 years and 2 cm between 12 and 13 years of age. The Tanner puberty stages correspond to S1P1 at 11 years 6 months, S3P2 at 12 years 11 months, and P2A1S3 at 13 years 5 months. Menarche occurred at 13 years 3 months. **c** Male patient with attenuated form of MPS II (Hunter) shows normal growth until the age of 3 months, followed by sharp declines in growth velocity (− 2 SD) until the age of 4. There is a slight amelioration between 4 and 6 years (− 1 SD) but a steady decline at 11 years (− 2 SD), at 13 years (− 3 SD), and at 15 years (− 4 SD) of age. There was no evidence of puberty at 15 years and 1 month. The onset of puberty at 15 years 9 months (testicle size 30–35 mm, borders 40–50 mm, absence of pubic hair, and very low levels of AMH). There were continued slow growth and an adult height of 154 cm measured at 23 years (target height of 176.5 cm), although diagnosis was made at 7 years of age. **d** Female patient with attenuated MPS IV (Morquio) shows normal growth until the age of 6, after which there is a decline in growth velocity (− 2 SD between 10 and 13 years). At 10 years 11 months, the mammary glands measured 4 cm. Menarche occurred at 13, but oligomenorrhea and marked growth deceleration ensued with a final adult height of 143 cm at 14 years (target height 161 cm). Diagnosis of Morquio was confirmed at 7 years. **e** Male patient with attenuated form of MPS VI (Maroteaux-Lamy) shows normal growth until the age of 1.5, followed by a slight but progressive decline in growth velocity (− 1 SD) at 2 years, followed by − 2 SD at 3.5 years and − 3 SD after age 9. There was no evidence of puberty at 14 years and an adult height of 154 cm measured at 19 (target height 182 cm). This patient was diagnosed at 12 years of age

Growth impairment and short stature are common features seen in all MPS patients, regardless of type or severity (Fig. 2a–e) [12, 15, 26, 37, 41]. The adult height attained depends on phenotype severity. In the attenuated forms, subtle growth deviations (− 1 standard deviation (SD) compared with a normative sex-specific growth chart) can begin as early as 9 months of age (Fig. 2a) with irregular, unbalanced, and decreasing growth velocities as the child gets older. What is notable is the significant growth deficits seen before the age of 12 years (between 5 and 12 years), delayed puberty, and insignificant or absent pubertal growth spurt, regardless of the AMPS type (Fig. 2a–e).

Disease-specific reference growth charts have been developed for several of the MPSs (II, IV, VI) [29, 37, 41]. However, very little growth data is available to describe longitudinal growth profiles for MPS I, except for several observational case series and mostly of mixed phenotypes [20, 21, 43, 55].

#### Growth impairment in the AMPS I (Hurler-Scheie and Scheie)

Growth in AMPS I (Hurler-Scheie and Scheie) patients is compromised at all ages and adult height ranges from 130 to 164 cm, depending on phenotype severity [20, 21, 47, 55]. Growth profiles of two female AMPS I patients (Hurler-Scheie and Scheie) are presented in Fig. 2a, b. Normal growth was measured until 9 months in one patient (Hurler-Scheie) and 1 year in the other (Scheie), after which both demonstrated irregular and decreased growth velocities. Growth deviations of – 2 SD and − 2.5 SD were measured at 9 and 10 years respectively before confirmation of Hurler-Scheie diagnosis at age 10, and before treatment initiation (Fig. 2a). For the second AMPS I patient (Fig. 2b), a diagnosis of Scheie was not confirmed until 20 years of age. For this patient, subtle growth deficits were noted at 3 years (− 1 SD) and at 4 years (− 1.5 SD) of age which sustained until the age of menarche at 13 years, after which there was growth cessation with patient attaining an adult height of 146 cm (− 3 SD). There was a suboptimal puberty growth spurt (+ 12 cm) between ages of 10 and 13 years as compared with sex-specific French growth reference charts [46].

#### Growth impairment in AMPS II (Hunter)

MPS II (Hunter) is X-linked recessive and predominantly affects male patients [7, 46]. These patients tend to be taller and heavier than unaffected individuals up to the ages of 5–8 years [18, 52]. However, the growth velocity can begin to decrease as early as 2 years leading to a height below the 3rd percentile by the age of 8; nearly all patients exhibit a growth deficit by the age of 12 years [36, 40, 43]. The adult height can range from 116 cm to as high as 170 cm, depending on phenotype severity [6, 33, 37, 44]. The patient with AMPS II represented in Fig. 2c was diagnosed at the age of 7. The growth profile illustrates a delayed puberty at 15 years 9 months, continued slow growth, and an adult height of 155 cm at 23 years.

#### Growth impairment in AMPS IV (Morquio)

In MPS IV, growth velocity and adult height are often used as indicators of disease severity [15, 29, 52]. In severe forms, growth stops at 7–8 years of age with an adult height of < 120 cm [52]. In attenuated forms, growth continues slowly into the teenage years and mean adult height is 113 ± 23.1 cm for females and 119 ± 22.6 cm for males [15, 29, 52]. The growth profile of the AMPS IV patient presented in Fig. 2d shows remarkably normal growth until the age of 6, then – 2 SD at 10 years and until menarche at 13 years. Oligomenorrhea and growth cessation ensued with an adult height of 143 cm (− 3 SD) at 14 years. Diagnosis of Morquio had been confirmed at 7 years.

#### Growth impairment in APMS VI (Maroteaux-Lamy)

Quartel et al. have developed and characterized growth charts specific for patients with AMPS VI given their different progressions of growth [41]. The adult height for patients with AMPS VI can range from 126 to 161 cm depending on disease severity [41, 51]. The growth profile of a male AMPS VI patient is presented (Fig. 2e). Normal growth is observed until age 1.5 years, after which there is a slight but progressive decline in growth velocity (− 2 SD at 3.5 years and − 3 SD after the age of 9). In this patient, diagnosis was confirmed at 12 years of age. The adult height was 154 cm at the age of 19.

#### Growth impairment in APMS VII (Sly syndrome)

Montano et al. published growth data on 12 patients with MPS VII [30]. No abnormalities were detected in patients before 2 years of age. The adult height measured was 151.18 ± 7.9 cm in males and 114.3 ± 25.4 cm in females.

### Underlying metabolic pathology

Suspicions of an underlying metabolic pathology should arise if a child suddenly stops growing or slowly and steadily changes growth trajectory. Although significant changes in growth velocity can occur as early as 3 years of age, children with AMPS are most often referred to an endocrinologist much later (at around 10 years) when there is delayed puberty (complete or incomplete), secondary amenorrhea, or endometriosis. What is certain and aberrant for all forms of AMPS is the suboptimal or absent pubertal growth spurt (2–5 cm as opposed to an anticipated 20–25 cm), which should immediately prompt further investigation into underlying metabolic disorders.

On examination, growth hormone and gonadal and thyroid hormonal functions in children with AMPS are found to be normal explaining why up to 50% of the cases are deemed idiopathic [22]. In these situations, the authors encourage the endocrinologist to request and review full-body radiological evaluations and assess the correlation between bone maturation and age of the child (Modified Oxford Scale) [48]. Late bone maturation (pseudo delayed bone age or delayed ossification of the bone) is observed in all forms of AMPS [38]. It is paramount to note that if MPS is diagnosed early and ERT initiated before puberty, the adult height could be improved [18, 20, 21, 47].

### Bone and joint involvement

The most suggestive features of many of the MPSs are dysostosis multiplex, skeletal dysplasia, and resulting short stature [26, 31, 54]. Abnormal bone ossification affects all bones and joints of the skeleton [26, 31, 49, 54]. The primary cause of chronic joint stiffness, limiting range of joint mobility (ROM) in patients with MPS (I, II, VI, and VII), and ligamentous laxity, responsible for joint hypermobility in MPS IV, is the involvement of GAG in the ligaments and capsule around the joint [28, 52]. All joints are affected but with varying rates of progression which ultimately result in degenerative joint disease if left untreated [27].

Limitations in ROM affect activities of daily living (ADL) including mobility and self-care, tend to increase with age, and are more pronounced in more severe forms of MPS [25]. In our experience, parents are among the first to notice joint anomalies, especially in the shoulders, hands, and knees. The child often expresses difficulty in lifting arms and moving shoulders in efforts to get dressed, expresses clumsiness in holding a pencil, or demonstrates foot rigidity (nonplantigrade feet) and has difficulty wearing shoes. Children also often express pain in their hips and back when walking.

Restricted joint function and ROM are noted on physical examination; there is limited extension of the fingers, elbows, shoulders, hips, and knees, whereas joint inflection is often preserved. Pediatricians should ask the parents whether the child has difficulty sleeping (due to pain) or difficulty with fine motor tasks (due to loss of sensitivity and numbness in hands) as this should raise suspicions of nerve compression and carpal tunnel syndrome which is frequently seen in MPSs I, II, and VI. We have noted that children with MPS complain minimally of pain: as such, diminished abduction and anteflexion in the shoulder (an indicator of stiffness) and flexion deformity of the elbow are often overlooked. Stiffness of the knees and ankles as well as knee and ankle flexion deformities inhibits the heel–sole–toe stance (the plantigrade foot) and can have a significant impact on gait and the ability to walk independently [27, 31].

In our experience, joint stiffness and ROM in the absence of inflammation are often the first presenting symptoms for which the patient is referred to a rheumatologist. In our opinion, many physicians tend to underestimate the importance of limited ROM. In practice, differential diagnoses are numerous, and AMPS are misdiagnosed as rheumatic fever or as atypical inflammatory rheumatoid arthritis (juvenile inflammatory arthritis if early onset), psoriatic arthritis, etc. [27, 31]. However, limited range of joint mobility, even moderate, in the absence of clinical or biological inflammation is signs of pathology (especially among children) and should raise suspicions of an MPS disorder [1, 7, 25].

#### Radiology and imagery

Characteristic bone abnormalities seen collectively, described as dysostosis multiplex, are typical in MPS patients, and radiologic imagery of anomalies has been described in the most severe cases [31, 35, 49, 54]. As would be expected, radiographic findings in AMPS patients are not as apparent as in severe forms of the disease; awareness of subtle radiologic anomalies is thus essential for the radiologist to suspect AMPS. We recommend that the rheumatologist or orthopedic physician assesses anteroposterior and lateral views of the spine, from the hip to ankle, and bilateral functioning of the hands, elbows, and shoulders in the diagnostic workup of any suspected case of MPS, regardless of severity.

**Fig. 3:**
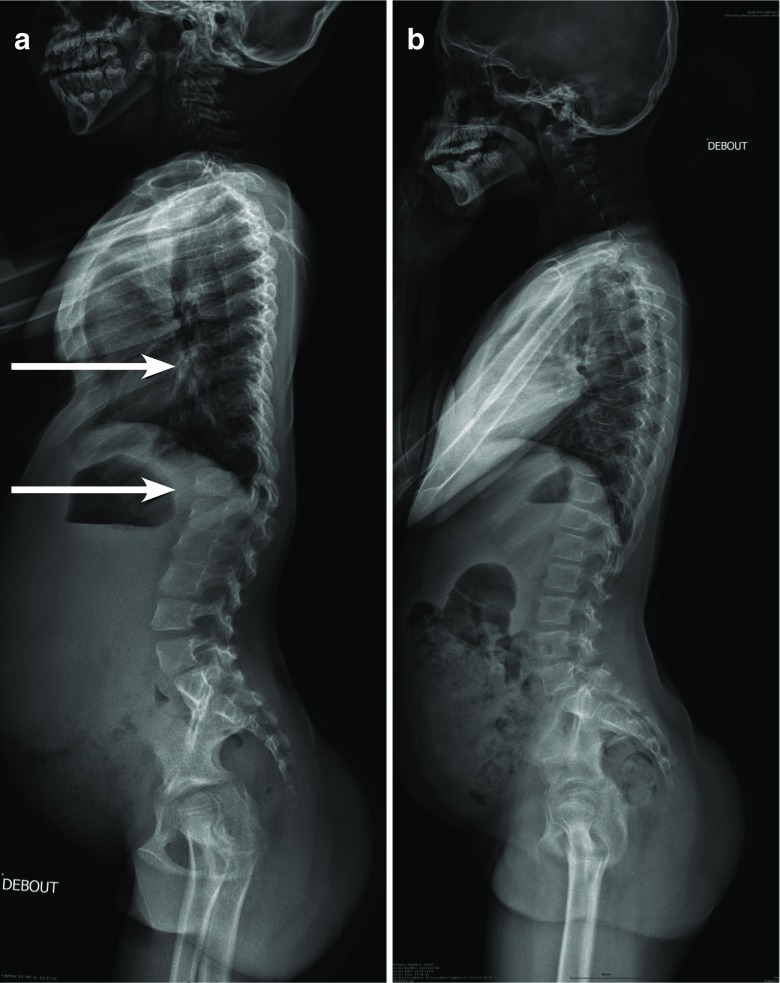
Radiographic image of the spine profile in a 7 year old child with attenuated form of MPS I (**a**). There is thoracic kyphosis in the thoracolumbar junction, but without the characteristic appearance of the beak-like projection generally seen on the L1 or L2. The vertebrae are discretely ovoid shape in the thoracic region. However, the same radiographic image of another child (11 years old) with attenuated MPS I does not show any anomalies (**b**)

It is our opinion that the most significant radiologic findings in AMPS patients are seen in imagery of the spine, pelvis, and hands: thoracolumbar kyphosis/scoliosis is extremely common in MPS patients [31, 35, 54]. In the more severe forms of the disease, hallmark features include rounded vertebral bodies, “anterior beaking” (generally seen on L1 or L2), posterior scalloping, and platyspondylia with wedge-shaped deformities [35]. However, in attenuated forms, there may be an absence of “anterior beaking,” and in some cases an absence of anomaly altogether, as shown in Fig. 3.

**Fig. 4:**
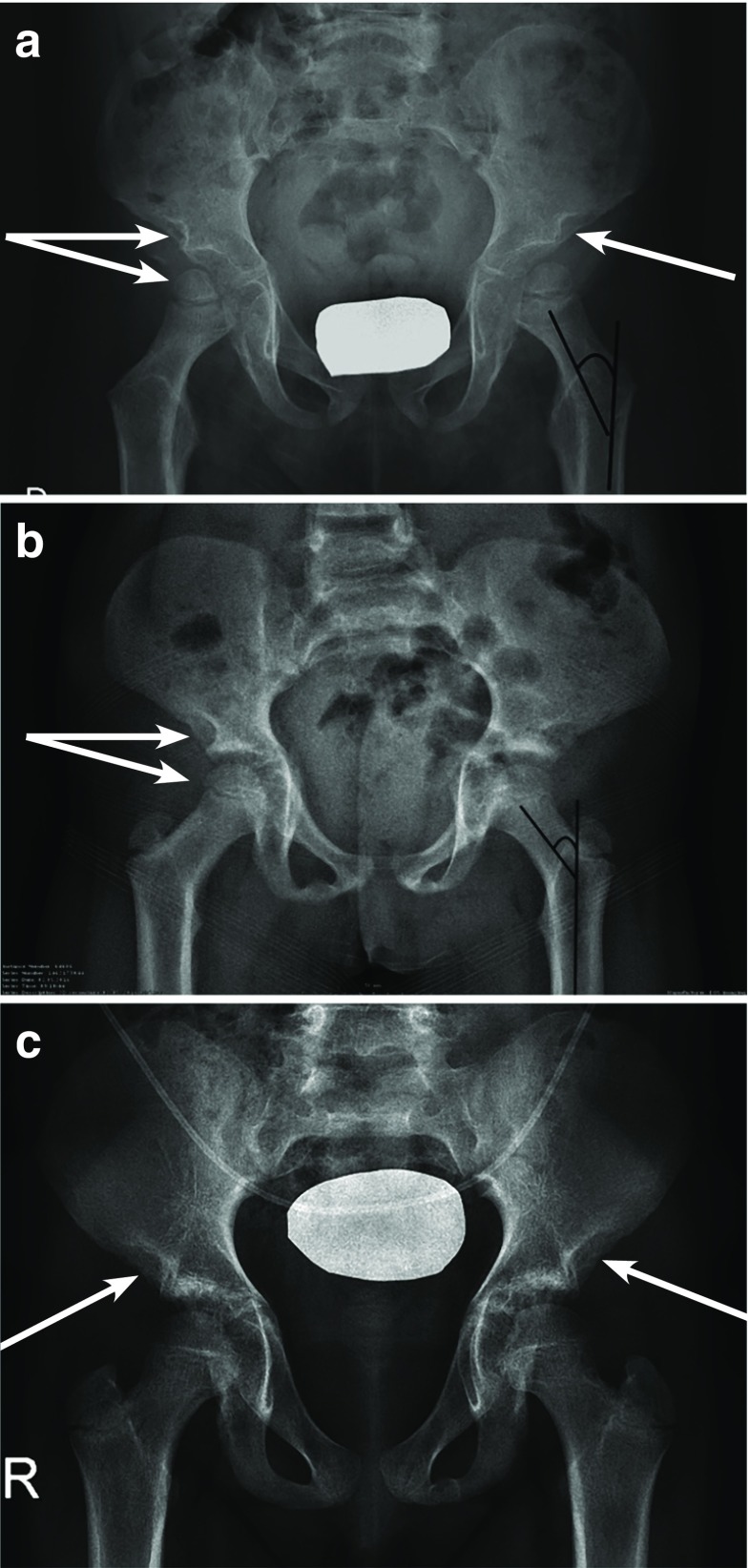
Frontal radiographic image of the pelvis in two children with attenuated MPS I (a 4-year-old (**a**) and an 8-year-old (**b**)), the anomalies are similar and associated with long and narrow femoral neck, a superior femoral epiphyses given the short stature, a relative coxa valga, a moderate acetabular dysplasia, and poorly developed acetabulum. Similarly, in a third child (age 12 years), all the anomalies mentioned are much more attenuated, with the exception of the characteristic of acetabulum which is noted in all radiographic images of attenuated MPS I (**c**)

**Fig. 5:**
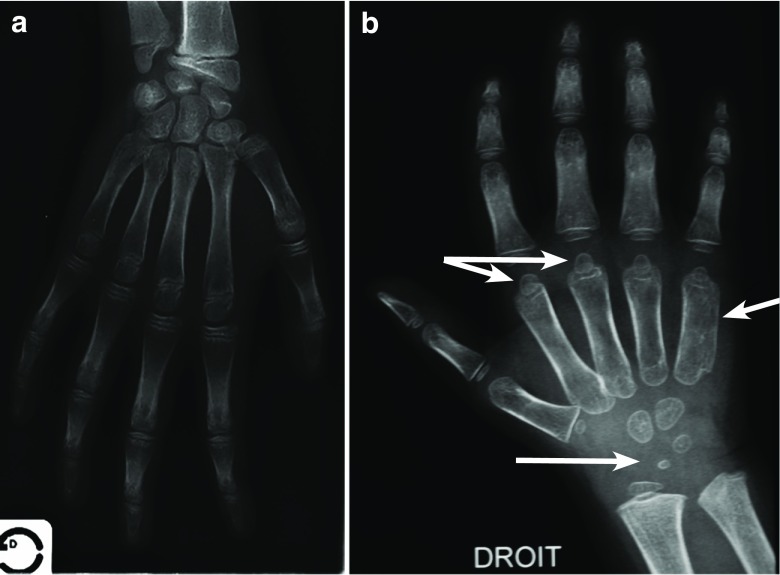
The bone anomalies of the hand are at times difficult to capture, generally very moderate (**a**) or at times absent (**b**). Short metacarpal epiphyses, a thickening of the metacarpals and sometimes the phalanges, a delayed ossification of the carpus with respect to the chronological age

Multiplex dysostosis of the pelvis in AMPS patients may include a narrowed femoral neck including relative coxa valga, small proximal femoral epiphysis, a moderate acetabular dysplasia, and an abnormal wedge-shaped acetabulum (Fig. 4). Epiphyseal dysplasia is commonly seen in the hands and wrists of patients with MPS regardless of severity [31, 35]. However, in AMPS patients, these anomalies are at times difficult to capture radiographically as they are moderate or even absent (Fig. 5). The metacarpals and sometimes the phalanx tend to be shorter and thicker than normal, and there is a delayed ossification of the carpus with respect to the chronological age [49].

### Multiple, concurrent, and other seemingly unrelated symptoms

There is often a myriad of unsuspecting, insidious, and non-specific signs which may or may not be apparent in routine physical examination or the child’s medical history: frequent ear, nose, and throat (ENT) infections; snoring and trouble sleeping; obstructive sleep apnea; decreased pulmonary function; hearing loss; etc. Hernias (inguinal and/or umbilical) in non-premature young children, or coarse facial features and corneal clouding, should also raise suspicions of AMPS I, IV, or VI [3, 7, 16].

Patients with AMPS are thus likely to undergo multiple, often unrelated, surgical procedures occurring at atypical ages, compared with the general pediatric population [1]. ENT surgery is the most frequent (e.g., tonsillectomy, adenoidectomy, and ear tubes). Other surgical interventions include tendon release for trigger fingers and carpal tunnel release, multiple hernia repairs, and, more rarely, cardiac valve replacement [1].

### Diagnostic challenges and measurement caveats

Many rheumatologists attribute pain and joint limitations in young children to growing pains, juvenile idiopathic arthritis, or other systemic rheumatic disorders. However, the absence of inflammation should prompt further radiologic workup with at least a profile view of spine and a front view of the pelvis and hand, as mentioned above [7].

It is well known that a child’s height and weight are important parameters for monitoring growth and well-being and that these parameters should be plotted on growth charts to follow a child’s growth trajectory. Any decrease in growth rates, especially during puberty, should alert the pediatrician to an underlying disorder. Nevertheless, the interpretation of growth trajectories should take into account the mid-parental stature and child’s growth potential [17].

## Consensus/conclusion

Here, experts from various pediatric specialties have developed an algorithm to help increase awareness of AMPS. Parents are often the first to notice the burgeoning symptoms, beginning as early as 2 to 4 years of age. Symptoms include slight but progressive decrease in growth velocity, limitations in joint mobility, loss of fine motor skills, and pain. Pediatricians and consulted specialists should engage parents in discussions of symptoms and symptom onset as they are the first and primary caretakers of children and their observations are key in promoting suspicions of the disorder. Pediatricians must also become familiar with the child’s medical history, especially with respect to a high frequency of ENT infections, hernias, frequent hospitalizations, and surgeries. Correctly measured height and weight should be plotted on growth curves with special attention to progressive decline in growth trajectories, and especially in the event of incomplete puberty, puberty delays, and suboptimal pubertal growth spurt. Young patients with joint limitations and/or idiopathic growth deficiencies should be further investigated early on with radiologic evaluations (of the hands, elbows, shoulders, hips, pelvis, and spine). Once an MPS disorder is suspected, uGAG testing and specific enzyme analyses should be done to confirm diagnosis, and the child referred to a metabolic specialist at a reference center to define an adapted therapeutic approach to optimize outcome.

## Abbreviations

ADL: Activities of daily living
AMPS: Attenuated mucopolysaccharidosis
ENT: Ear, nose, and throat
ERT: Enzyme replacement therapy
GAGs: Glycosaminoglycans
MPS: Mucopolysaccharidosis
ROM: Range of motion
SD: Standard deviation
